# The Role of Ultrasound in the Evaluation of Tardive Dyskinesia: A Case Series

**DOI:** 10.5334/tohm.966

**Published:** 2025-01-17

**Authors:** Ujjawal Roy, Ajay Panwar, Achal Kumar Srivastava, Michael S. Cartwright

**Affiliations:** 1 Department of Neurology, Pulse Superspeciality Hospital, Ranchi, India; 2 Department of Neurology, Rotary Ambala Cancer and General Hospital, Ambala, India; 3 Department of Neurology, All India Institute of Medical Sciences, New Delhi, India; 4 Department of Neurology, Wake Forest School of Medicine, Winston-Salem, NC, USA

**Keywords:** Tardive syndrome, Tardive Dyskinesia, tardive dystonia, Ultrasonography, ultrasound guidance

## Abstract

**Background:**

Despite efforts to visualize all the movements of tongue and oropharynx in individuals with focal movement disorders (specifically tardive dyskinesia (TD)), clinicians can miss the complete picture and additional tools may be required to reach an accurate diagnosis.

**Cases:**

We present three cases with TD where ultrasound assisted in diagnoses. These individuals had difficulty swallowing and abnormal sensations in the tongue, which remained undiagnosed until we performed ultrasound of oropharynx which allowed for characterization of these movements.

**Discussion:**

Ultrasound is an ideal modality for imaging the tongue and oropharynx in TD. Further research should include large case series and standardized protocols for evaluation of these disorders.

## Introduction

Tardive dyskinesia (TD) is an umbrella term used to encompass dystonia, chorea, akathisia, and other movement disorders, and it is typically caused by long-term use of dopamine receptor blocking agents [1]. As the diagnosis of these disorders is clinical, it is possible to miss the complete phenomenology if one is not able to visualize all components of the tongue and oropharynx. Here we discuss three cases of TD where ultrasound aided in identification of specific movements of the tongue and pharyngeal muscles, thus contributing to a holistic approach to diagnosis and management.

## Case Series

### Case 1

**Figure 1:**
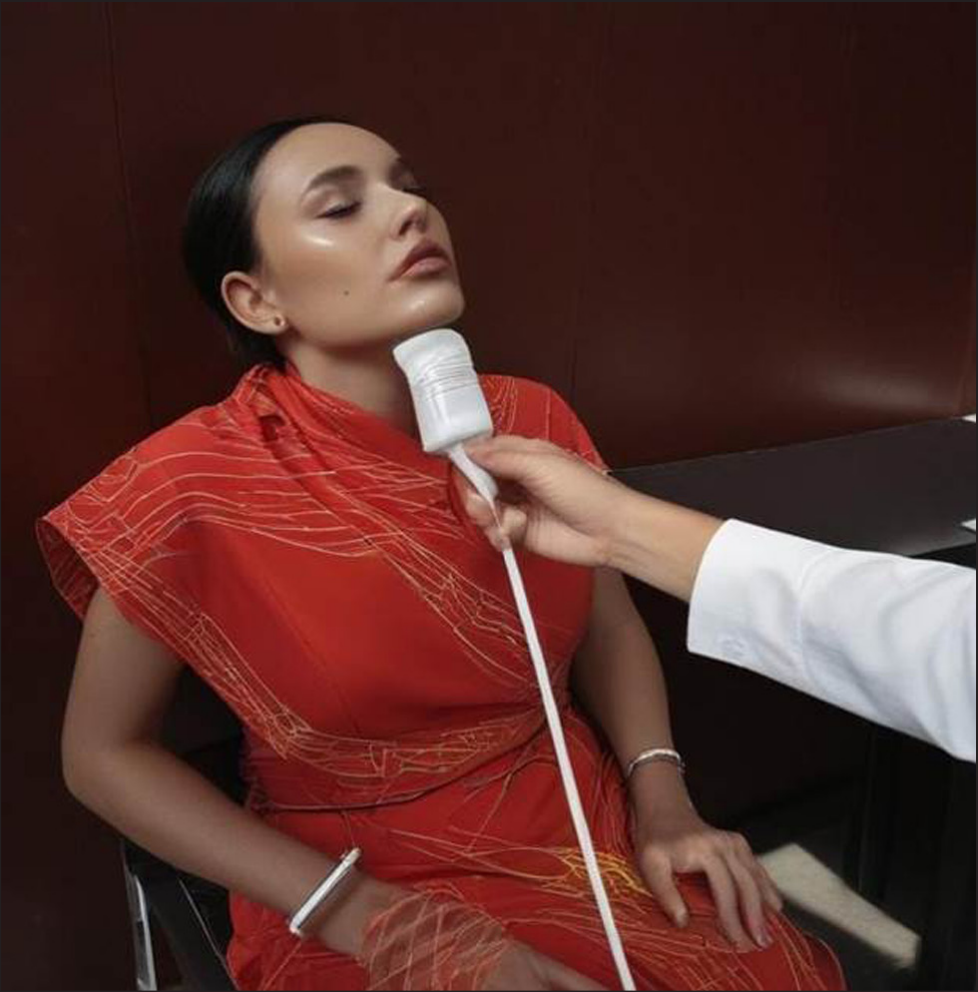
Shows the ultrasonography technique of submental view with the linear probe.

A 38-year-old woman presented with 8 months of difficulty chewing and swallowing, as well as abnormal sensations in her tongue, especially with her mouth closed. The tongue had no abnormal movements on visual examination. She had visited different specialists, including otorhinolaryngology, dentistry, and psychiatry, and she was prescribed antidepressants and antacids. She did not experience any relief in her symptoms, after which she was referred to us. Her physical examination included careful visual inspection of tongue, which showed no abnormalities (Video 1). All laboratory parameters including peripheral smear for acanthocytes and magnetic resonance imaging (MRI) of the brain were normal. In view of persistent complaints pertaining to her oral cavity when her mouth was closed, we decided to perform ultrasonography of the tongue during mouth closure using a Venue Go R4 (GE HealthCare, USA) with a 4–20 MHz linear transducer (submental view) (Figure 1). Ultrasound revealed dystonic contractions of the geniohyoid and genioglossus muscles (Video 2). On repeat enquiry, she disclosed the use of a drug for her “functional dyspepsia” for around 1.5 years, which contained levosulpiride. She had stopped that drug only one month previously. Hence, the diagnosis of TD was established. She was given the option of local botulinum toxin injection in the tongue. Botulinum toxin was injected at 2 sites of each genioglossus, starting with 5 U and escalating to 10 U in 2 cycles. Additionally, we injected 2 sites of each geniohyoid also, starting with 2.5 U and escalating to 5 U. At 4 month follow up (after the second cycle) she reported marked improvement in her symptoms. The abnormal sensations in her tongue had markedly decreased and she was able to swallow normally, and ultrasound of the tongue showed a marked decrease in the dystonic movements (Video 3).

**Video 1:** Shows visual inspection of tongue, which did not reveal any abnormality.

**Video 2:** Shows ultrasonography of tongue which revealed dystonic contractions of geniohyoid muscle (arrow from above) and genioglossus muscle (arrow from below).

**Video 3:** Shows ultrasonography of tongue after botulinum toxin injection which revealed marked improvement in the dystonic contractions of the muscles.

### Case 2

A 36-year-old woman presented with 8 months of abnormal movements of her face and tongue. She complained of dysphagia, which she attributed to the abnormal tongue movements during chewing and swallowing. She had a history of escitalopram 10 mg and haloperidol 3 mg use on a regular basis for one year. Accordingly, she was diagnosed with the orofacial and lingual form of TD, and she was withdrawn from the medications. Additionally, she received injections of botulinum toxin in the tongue, however, the exact dose and sites of injection were not available. She did not experience any improvement in her symptoms; rather there was a subjective exacerbation of the dysphagia. On examination there were dystonic movements noted in her face (orbicularis oris, mentalis, and corrugator supercilii), additionally we observed subtle dyskinetic movements of the pharyngeal region (Video 4). All other investigations, including MRI head and labs, were normal. Tongue ultrasound revealed dyskinetic movements of the pharyngeal region, disproportionately more than that of the tongue and orofacial regions (Video 5). We counseled the patient regarding the disease and added tetrabenazine, clonazepam, and baclofen. We planned for botulinum toxin injection focused on the pharyngeal muscles and face. At 1 month follow up she had symptomatically improved with respect to the dysphagia as well as orofacial dystonia (Video 6). Ultrasonography of the tongue also showed marked improvement in the dyskinetic movements in the pharyngeal region (Video 7).

**Video 4:** Shows dystonic movements noted in the region of face (orbicularis oris, mentalis, and corrugator supercilii) along with subtle dyskinetic movements of the pharyngeal region.

**Video 5:** Shows ultrasonography of tongue and pharynx which revealed dyskinetic movements of the pharyngeal region (arrow), disproportionately more than any other region.

**Video 6:** Shows marked improvement in dystonic contractions of the orofacial region after initiation of the treatment.

**Video 7:** Shows ultrasonography of tongue and pharynx revealing near resolution of dyskinetic movements of the pharyngeal region after initiation of the treatment.

### Case 3

A 32-year-old man presented with 6 months of difficulty speaking, involuntary jaw closure, and frowning with excessive eye blinking while speaking and eating, along with abnormal sensations of his tongue, particularly when his mouth was closed. He had a history of risperidone (4 mg/day) use for the last 4 years and had persistence of these symptoms even after tapering off the medication two months prior. On visual inspection there was jaw closure dystonia, frontalis and corrugator supercilii dystonia, and blepharospasm. However, visual inspection of the tongue showed no abnormalities (Video 8). All other investigations including MRI and labs were normal. Ultrasound of the tongue revealed dystonic contractions of the geniohyoid and genioglossus muscles (Video 9). He was diagnosed with TD and given the option of local botulinum toxin injection. He opted for trihexyphenidyl (building up to 4 mg/day), baclofen, and clonazepam and has not yet been seen in follow-up.

**Video 8:** Shows visual inspection revealing jaw closure dystonia, frontalis and corrugator supercilii dystonia/dyskinesia, and blepharospasm without any abnormality of the tongue.

**Video 9:** Shows ultrasonography of tongue which revealed frank dystonic contractions of the geniohyoid muscle (arrow) and subtle contractions of genioglossus muscle.

## Discussion

TD has a wide array of phenomenology and different body parts may be involved, which are at times subtle. Additionally, some movements have distinct locations which can be missed if the diagnosis is only based on visual inspection [1]. Hence, additional tools are welcomed for further investigating individuals with suspected TD.

In recent times, point of care ultrasonography (POCUS) which is both performed and interpreted by the clinician, has emerged as a rapid diagnostic tool and become increasingly popular as the clinician can corroborate the sonographic findings with history and clinical examination at the patient’s bedside [2]. Recently, POCUS has been used in neurology extensively in the evaluation of many diseases [3]. However, tongue ultrasonography using the submental approach has been limited to tumour staging, vascular malformations, evaluation of fasciculations, and a few other indications [4]. Although in rare instances it has been used as an adjunct to clinical examination [5], its importance in the evaluation of suspected TD has not been highlighted to date.

We used ultrasound to assess all the three patients discussed above with orolingual and pharyngeal dyskinesias, and ultrasound greatly assisted with the diagnosis and management of these cases. Position and task-specificity aggravating dystonia while speaking and during mouth opening has been reported previously, and it is generally easy to visualize [5]. Conversely, our first and third cases only had abnormal tongue movements upon closure of the mouth, and the movements were bound to be missed otherwise. Additionally, isolated tongue dystonia has been rarely reported in the literature, so it is not a common finding in TD [6]. Hence, the main advantage of ultrasound in these cases was that it was able to assess the tongue with the mouth closed. Although electromyography could aid in the diagnosis of tongue dystonia [7], it cannot be used in all patients, as it can be difficult to interpret in addition to being invasive and painful. Additionally, researchers have concluded that in cases of dystonia of the tongue, especially those with a “retraction type,” patients often cannot reproduce dystonic contraction during EMG examination, hence it is difficult to detect the true muscles causing the dystonic symptoms [8], which could be clearly seen by ultrasonography in our cases. This could determine the exact site and muscle for botulinum toxin injection as in our cases, which resulted in excellent results both clinically as well as ultrasonographically as opposed to unsatisfactory results reported by previous researchers in cases of retraction dystonia [8]. The treatment of dystonia has significantly improved with the advent of botulinum toxin, as it is able to selectively target muscles with the help of ultrasound, as opposed to oral treatments which are systemic and may have side effects [9,10]. Ultrasound was also able to assess the pharyngeal region accurately, which assisted in refining the diagnosis in the second case. It is likely in this case that since the previous treatment paradigms were based only on visual examination, outcomes were incomplete and unsatisfactory. Furthermore, although it is common in TD to have multiple dyskinesias and dystonias, including the face, tongue, and neck [1], pharyngeal dystonia has been rarely reported [11] and is easily missed when there are multiple dyskinesias of the orolingual and facial regions. It is important to note that this case had not only involvement of the pharyngeal region but also of the upper face, which is classically seen in Huntington’s disease [12].

Development of additional tools such as ultrasound for the assessment and diagnosis of disorders such as dystonias of the orolingual and pharyngeal regions is key, as the diagnosis can be missed if it is only based on visual inspection. Ultrasound is an ideal imaging modality for these cases because it is high resolution and can accurately depict real-time movements. Ultrasound is feasible to use at the bedside and in outpatient clinics, is cost-effective, and it allows for repeat imaging, which helps in follow-up after initiation of treatment. In the past, researchers have emphasized the role of ultrasonography in obtaining anatomical information regarding muscle hypertrophy and atrophy, side to side asymmetries, and other anomalies, which facilitates the safe administer of botulinum toxin in individuals with cervical dystonia [13]. Naturally, this model is applicable to orolingual and pharyngeal regions as well but has not been used in lingual dystonia [14]. Ultrasound can be a powerful tool not only to diagnose but also to quantify different movements in different muscles in various disorders [15], and potentially can aid in chemodenervation in complex cases. However, ultrasound has a few limitations. It is operator dependent and formal training is prudent prior to imaging the oropharyngeal muscles. Nevertheless, bedside ultrasound can be a third eye for clinicians, and is hence gaining popularity worldwide.
